# Effects of Exercise on Progranulin Levels and Gliosis in Progranulin-Insufficient Mice^1^,^2^,^3^

**DOI:** 10.1523/ENEURO.0061-14.2015

**Published:** 2015-07-03

**Authors:** Andrew E. Arrant, Aashka R. Patel, Erik D. Roberson

**Affiliations:** Departments of Neurology and Neurobiology, Center for Neurodegeneration and Experimental Therapeutics, University of Alabama at Birmingham, Birmingham, Alabama

**Keywords:** BDNF, exercise, frontotemporal dementia, progranulin

## Abstract

Loss-of-function mutations in progranulin (*GRN*) are one of the most common genetic causes of frontotemporal dementia (FTD), a progressive, fatal neurodegenerative disorder with no available disease-modifying treatments. Through haploinsufficiency, these mutations reduce levels of progranulin, a protein that has neurotrophic and anti-inflammatory effects. Increasing progranulin expression from the intact allele is therefore a potential approach for treating individuals with *GRN* mutations. Based on the well-known effects of physical exercise on other neurotrophic factors, we hypothesized that exercise might increase brain progranulin levels. We tested this hypothesis in progranulin heterozygous (*Grn^+/−^*) mice, which model progranulin haploinsufficiency. We housed wild-type and progranulin-insufficient mice in standard cages or cages with exercise wheels for 4 or 7.5 weeks, and then measured brain and plasma progranulin levels. Although exercise modestly increased progranulin in very young (2-month-old) wild-type mice, this effect was limited to the hippocampus. Exercise did not increase brain progranulin mRNA or protein in multiple regions, nor did it increase plasma progranulin, in 4- to 8-month-old wild-type or *Grn^+/−^* mice, across multiple experiments and under conditions that increased hippocampal BDNF and neurogenesis. *Grn^−/−^*mice were included in the study to test for progranulin-independent benefits of exercise on gliosis. Exercise attenuated cortical microgliosis in 8-month-old *Grn^−/−^*mice, consistent with a progranulin-independent, anti-inflammatory effect of exercise. These results suggest that exercise may have some modest, nonspecific benefits for FTD patients with progranulin mutations, but do not support exercise as a strategy to raise progranulin levels.

## Significance Statement

Haploinsufficiency of progranulin causes frontotemporal dementia, and strategies to increase progranulin expression from the intact allele could have a therapeutic benefit. Here we show that voluntary wheel running under conditions that are sufficient to increase hippocampal BDNF and neurogenesis does not increase brain or plasma progranulin levels in 4- to 8-month-old wild-type or *Grn^+/−^*mice. However, exercise reduced cortical microgliosis in *Grn^−/−^*mice, indicating an anti-inflammatory effect independent of progranulin. These data indicate limited benefits of exercise in the progranulin-insufficient mouse model of frontotemporal dementia without normalization of progranulin levels.

## Introduction

Frontotemporal dementia (FTD) is a devastating neurodegenerative disorder with multiple clinical subtypes and genetic causes (Mackenzie et al., 2010; Karageorgiou and Miller, 2014). In the most common FTD subtype, behavioral variant FTD, patients present with social dysfunction, apathy, disinhibition, repetitive behavior, and other behavioral abnormalities (Rascovsky et al., 2011; Karageorgiou and Miller, 2014). FTD has a strong genetic component, with 10–25% of patients having dominantly inherited FTD (Stevens et al., 1998; Ratnavalli et al., 2002; Goldman et al., 2005; Seelaar et al., 2008; Rohrer et al., 2009).

Loss-of-function mutations in *GRN*, the progranulin gene, are one of the most common genetic causes of FTD and account for 5–10% of all FTD cases (Baker et al., 2006; Cruts et al., 2006; Gass et al., 2006). Most mutations cause haploinsufficiency of progranulin, a secreted glycoprotein that is expressed throughout the body, and in the brain is expressed by neurons and microglia (Bateman and Bennett, 2009; Petkau et al., 2010). Progranulin-insufficient mice (*Grn^+/−^* and *Grn^−/−^*) provide an animal model of progranulin haploinsufficiency and have several phenotypes that may model aspects of FTD. Both *Grn^+/−^* and *Grn^−/−^* mice develop abnormal behavior and show signs of amygdala dysfunction beginning around 6 months of age (Kayasuga et al., 2007; Yin et al., 2010; Ghoshal et al., 2012; Filiano et al., 2013). *Grn^−/−^*, but not *Grn^+/−^* mice, develop progressive gliosis, inflammation, and lipofuscinosis in several brain regions that first becomes detectable around 6–7 months of age and is more strongly elevated by 12 months (Ahmed et al., 2010; Yin et al., 2010; Wils et al., 2012; Filiano et al., 2013; Götzl et al., 2014).

The lack of disease-modifying treatments is a major hurdle facing FTD patients. Because almost all FTD-related *GRN* mutations produce progranulin haploinsufficiency, boosting progranulin levels is a logical approach to the prevention or treatment of FTD due to *GRN* mutations. Efforts are underway to pharmacologically increase progranulin (Cenik et al., 2011; Lee et al., 2014), but nonpharmacologic interventions may also be effective. In particular, physical exercise was reported to increase hippocampal progranulin levels in wild-type mice (Asakura et al., 2011). This is consistent with the well-known neurotrophic effects of exercise, which include increases in hippocampal BDNF and hippocampal neurogenesis (Neeper et al., 1995; van Praag et al., 1999a,b). Exercise may also be beneficial for FTD through mechanisms not directly related to progranulin expression, insofar as exercise reduces inflammation and stimulates autophagy, both of which could be protective in FTD (Gleeson et al., 2011; He et al., 2012a,b).

In keeping with these beneficial effects, data from humans and animal models indicate that exercise may be a valuable tool for preventing and treating neurodegenerative disorders. The benefits of exercise are most clear for Alzheimer’s disease (AD), though studies also show benefits for unspecified dementia. Physical activity or physical fitness are associated with lower risk or later onset of AD and other dementias, and exercise may slow cognitive decline and improve quality of life in patients with AD or unspecified dementias (Laurin et al., 2001; Buchman et al., 2012; Defina et al., 2013; Forbes et al., 2013; Winchester et al., 2013; Yu et al., 2015). Additionally, in animal models of AD exercise improves performance on memory tasks and reduces AD-like pathology (Stranahan et al., 2012; Intlekofer and Cotman, 2013).

It is currently unknown whether exercise could have similar benefits for FTD as for AD. Exercise-stimulated hippocampal neurogenesis and memory improvements directly address AD-related deficits, but this may not be as helpful in FTD, which is not primarily a disorder of memory. However, the anti-inflammatory and autophagy-stimulating effects of exercise could be beneficial for FTD. We therefore included *Grn^−/−^* mice in the study to test the secondary hypothesis that exercise could improve gliosis independent of any effect on progranulin expression.

In this study, we tested the hypothesis that exercise would normalize progranulin deficiency in *Grn^+/−^* mice. We provided mice with access to running wheels for 4 or 7.5 weeks. We hypothesized that this would be sufficient to produce stable increases in progranulin levels in the hippocampus of *Grn^+/−^* mice, but were also interested in whether progranulin might be increased in more FTD-relevant regions, such as the frontal cortex. This duration of wheel running is in the range of wheel running protocols that have produced improvements in mouse models of AD (Stranahan et al., 2012; Intlekofer and Cotman, 2013). We therefore anticipated some improvement of gliosis in *Grn^−/−^* mice.

## Materials and Methods

### Animals

**Figure 1:**
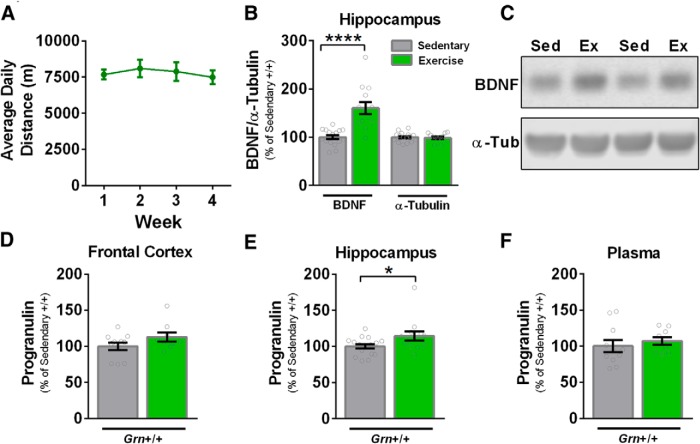
Exercise (3-4 weeks) produces a small increase in hippocampal progranulin in young adult wild-type mice. Two- to 3-month-old wild-type mice were randomized to sedentary or exercise groups for three to four weeks (***A***). ***B***, Exercise produced a robust increase in BDNF (****p < 0.0001). ***C***, Representative BDNF and α-tubulin blots. ***D***, Exercise did not significantly increase frontal cortex progranulin protein. ***E***, Exercise produced a small but statistically significant increase in hippocampal progranulin (*p = 0.038). ***F***, Exercise did not affect plasma progranulin. *n* = 8–16 mice per group. Values in ***B***–***F*** are expressed relative to the sedentary group.

The progranulin-insufficient mice used for this study were bred from a line generated by crossing *Grn*-floxed mice with mice carrying β-actin Cre, which were subsequently back-crossed onto a C57BL/6J background (Martens et al., 2012; Filiano et al., 2013). The entire coding region of the progranulin gene was deleted in the null allele. Wild-type littermates were used as controls for *Grn^+/−^* and *Grn^−/−^* mice. Both male and female mice were used. For the experiment with young adult mice (Fig. 1), 6-week-old male C57BL/6J mice were purchased from The Jackson Laboratory and housed in our animal facility for 2 weeks before beginning the exercise experiment. Wild-type (C57BL/6J background) male and female mice bred in our animal facility were used for the solo- versus group-housing experiment. All mice were housed in our Association for Assessment and Accreditation of Laboratory Animal Care-accredited barrier facility on a 12 h light/dark cycle, and given *ad libitum* access to food (NIH31 Diet, Harlan no. 7917) and water. All experiments were approved by the Institutional Animal Care and Use Committee at the authors’ university.

### Exercise

Mice were split into exercise or sedentary groups. Mice in the sedentary group were single-housed in a standard mouse cage, whereas mice in the exercise group were single-housed in a cage with an exercise wheel (Lafayette Instrument). For the experiment assessing effects of group-housing, mice were housed two to three per cage in each condition. During the experimental period, the exercise group was given 24 h access to running wheels, and running distance was recorded with software from the manufacturer (Lafayette Instrument). Mice in both groups were given *ad libitum* access to food and water throughout the experiment.

### Tissue Collection

At the end of the exercise period, mice were anesthetized with pentobarbital (100 mg/kg Fatal Plus, Vortech Pharmaceuticals). Blood samples were obtained by cardiac stick in a syringe containing 250 mm EDTA to prevent clotting. The blood samples were later centrifuged at 5000 x *g* for 15 minutes at 4°C for separation of plasma. The mice were transcardially perfused with 0.9% saline and the brains were collected and bisected. The left hemibrain was fixed in 4% paraformaldehyde for 48 h for immunohistochemical analysis, and the right hemibrain was frozen and stored at −80°C for biochemical analysis.

### Measurement of Progranulin Levels

For technical and experimental reasons, multiple techniques were used to measure progranulin levels. Progranulin mRNA and protein were measured in different brain regions of the experimental groups in an effort to determine if progranulin expression was increased, or if there was a slowing of progranulin degradation that would increase protein levels. Due to the different methods of sample preparation for mRNA or protein quantitation, mRNA and protein data are not available from the same tissue samples within a single experimental group. In all cases, progranulin mRNA was measured with RT-PCR. Progranulin protein levels were quantitated by two methods, depending on the sample type. Brain progranulin protein levels were measured in all cases by ELISA, and plasma progranulin protein was measured by western blot. Progranulin values were generally normalized to the wild-type sedentary control group, except in Figure 4, which included only *Grn^+/−^*mice. In this case, the exercised *Grn^+/−^*group was normalized to the sedentary *Grn^+/−^*group, which was set at 0.5 to maintain the same scale as in all other figures. All methods are described below in further detail.

### Progranulin ELISA

Brain progranulin protein levels were analyzed with an ELISA kit (Adipogen) using the manufacturer’s protocol. Hippocampus or frontal cortex samples were homogenized in lysis buffer (10 mm Tris, pH 7.5, 10 mm NaCl, 3mm MgCl_2_, 1mm EDTA, and 0.05% NP-40) with protease inhibitors (Halt protease inhibitor cocktail, Thermo Scientific) and protein concentration was determined by Bradford assay (Coomassie Plus, Thermo Scientific). The samples were diluted to 1 mg protein/ml with lysis buffer, and then diluted 1:1 with ELISA sample buffer. Samples were run in duplicate, with 50 µg of protein loaded per well. The progranulin content was calculated using a standard curve of recombinant progranulin from the same plate, and was normalized to the amount of protein loaded per well.

### Western Blots

Aliquots of the hippocampal lysates prepared as described above were analyzed by western blot for BDNF. The samples were diluted with LDS buffer (Life Technologies) and sample reducing agent (final concentration 50 mM dithiothreitol, Life Technologies), and then heated at 70°C for 10 minutes. The samples were then run on 4–12% bis-tris gels (Life Technologies) in MES buffer, with 20 µg of protein per lane. The gels were transferred to Immobilon-FL PVDF membranes (EMD Millipore), and the membranes were blocked for 1 h in 50% Odyssey blocking buffer (LI-COR Biotechnology). The membranes were probed for BDNF with an overnight incubation in rabbit monoclonal anti-BDNF antibody (1:500, Abcam EPR1292) diluted in 50% Odyssey blocking buffer. The next day, the membranes were incubated for 1 h in an IRDye 800-conjugated anti-rabbit antibody (1:20,000, LI-COR Biotechnology) and scanned on an Odyssey Scanner (LI-COR Biotechnology). After scanning, the membranes were stripped and reprobed for α-tubulin using a mouse monoclonal antibody (Sigma-Aldrich). α-Tubulin was detected with an IRDye 700-conjugated anti-mouse antibody (1:20,000, LI-COR Biotechnology). Bands were quantitated using Image Studio Lite software (LI-COR Biotechnology).

Plasma progranulin samples were analyzed by the above protocol with slight modifications. Plasma was diluted in RIPA buffer (50 mm Tris, pH 7.5, 150 mm NaCl, 5 mm EDTA, 0.1% SDS, 0.1% Triton X100, 0.5% sodium deoxycholate) with protease inhibitors, LDS buffer (Life Technologies), and sample reducing agent (Life Technologies), so that 2 µL of plasma were run per lane. After electrophoresis, transfer, and blocking as described above, the samples were incubated overnight in a sheep polyclonal anti-progranulin antibody (1:5000, R&D Systems AF2557). The following day, the membranes were incubated in a biotinylated anti-sheep antibody (1:2500, Vector Laboratories), followed by IRDye 800-conjugated streptavidin (1:1000, LI-COR Biotechnology). The membranes were then scanned and analyzed as described above.

### Immunohistochemistry

Brains were cut into 30 µm coronal sections on a sliding microtome (Leica) and the free-floating sections were used for immunostaining. The sections were blocked in 1% milk, 10% normal goat serum (Vector Laboratories), and 0.2% gelatin prior to overnight incubation in primary antibody. GFAP (1:5000 mouse monoclonal; Sigma-Aldrich G3893) or CD68 (1:500 rat monoclonal; AbD Serotec MCA1957) were diluted in 3% normal goat serum with 0.2% gelatin. Sections stained for doublecortin were blocked in 0.2% gelatin, and the primary antibody for doublecortin (1:250 goat polyclonal, Santa Cruz Biotechnology sc-8066) was diluted in 0.2% gelatin. The sections were then washed and incubated in the appropriate biotinylated secondary antibodies (1:1000 Vector Laboratories) for 1 h, followed by a 1 h incubation with avidin-biotin complex (Vectastain Elite, Vector Laboratories). Immunostaining was detected with diaminobenzidine (Sigma-Aldrich). Sections were then mounted on slides and coverslipped with Cytoseal 60 (Thermo Scientific). Slides were imaged on an upright microscope (Nikon), and two images per brain region, per mouse were analyzed for density of GFAP or CD68 immunostaining or for the number of doublecortin-positive neurons. Density was determined by thresholding images with ImageJ software and determining the percent of total image area containing GFAP- or CD68-positive pixels. Doublecortin-positive neurons were counted using ImageJ’s cell counter function. Lipofuscin granules were detected by epifluorescent imaging of tissue sections on an upright microscope (Nikon) in the green channel. The sections were counterstained with DAPI to provide anatomical landmarks. The density of autofluorescence was measured with ImageJ as described above.

### qRT-PCR

RNA was isolated from frozen cortex and thalamus samples with Trizol reagent (Life Technologies). The samples were treated with DNase to remove any contaminating genomic DNA (DNA-*free* kit, Life Technologies), and cDNA was generated with SuperScript III reverse transcriptase (Life Technologies). Taqman assays (Life Technologies) for *Tnfa* (Mm00443260_g1), *Ccl2 (Mcp1)* (Mm00441242_m1), *Il6* (Mm00446190_m1), and *Grn* (Mm01245914_g1) were used to analyze gene expression using the ΔΔ*C_t_* method. Expression of all genes was normalized to expression of *Actb* (Mm00607939_s1). qPCR was performed on a Roche LightCycler 480 using LightCycler 480 probes master mix (Roche).

### Statistics

Each dataset was first tested for normality and all were normally distributed. Running distance in progranulin-insufficient mice was analyzed by two-way repeated-measures ANOVA. Progranulin levels, BDNF, and the number doublecortin-positive neurons in progranulin-insufficient mice and solo/group-housed mice were analyzed by two-way ANOVA with genotype (or housing) and exercise as factors. Progranulin and BDNF were analyzed by *t* test in young adult mice and in the 4 week *Grn^+/−^*running experiment. Pathology data were analyzed by three-way repeated-measures ANOVA with genotype, exercise, and brain region as factors. For all analyses, significant interactions in higher-order ANOVA were followed by lower-order ANOVA for each brain region. Significant main effects or interactions in two-way ANOVA were followed by Sidak’s *post hoc* test to compare sedentary versus exercise mice of each genotype. Three-way ANOVA was conducted with JMP Pro 10 (SAS), and two-way ANOVA and *t* tests were performed with GraphPad Prism 6 (GraphPad Software). For all analyses, α was set at 0.05.

### Power Analysis

Each comparison made in this study was analyzed by power analysis using “Java applets for power and sample size” available at http://homepage.stat.uiowa.edu/~rlenth/Power/(Lenth, 2006-2009). The “two-sample *t* test” function was used for all calculations, with the critical comparison in each study used to calculate power. In all cases, the sample sizes and standard deviations from the actual data were used to calculate power for two-tailed comparisons of the control and exercise groups.

To aid interpreting the power of each comparison, particularly the negative data, we calculated several measures of power (see Table 1). The power of each experiment to detect a theoretical 30% change versus controls was reported to provide a measure of the ability to detect a moderate effect of exercise (as described below, a 30% change would be enough to bring progranulin in *Grn^+/−^* mice up to the lower limit of normal in wild-type mice). For measures of gliosis in *Grn^−/−^* mice, we also reported the power to detect normalization of gliosis. In addition, we calculated the change from control that each experiment could detect with powers of 0.7 and 0.8 to provide a measure of the smallest changes detectable with reasonable power. Figures are referenced below with the matching row from the statistical table noted with a superscript letter.

**Table 1:** Measures of progranulin levels

Row	Figure	Panel	Description	Test	Power for 30% change	70% power to detect change of:	80% power to detect change of:
**Measures of progranulin levels**							
a	1	D	Frontal cortex progranulin	Student's *t* test	0.93	22%	24%
b	1	E	Hippocampus progranulin	Student's *t* test	0.98	18%	21%
c	1	F	Plasma progranulin	Student's *t* test	0.82	26%	29%
d	3	A	Frontal cortex progranulin mRNA	Two-way ANOVA, Sidak's *post hoc* test	1	5%	6%
e	3	B	Thalamus progranulin mRNA	Two-way ANOVA, Sidak's *post hoc* test	1	8%	9%
f	3	C	Hippocampus progranulin	Two-way ANOVA, Sidak's *post hoc* test	1	11%	12%
g	3	D	Plasma progranulin	Two-way ANOVA, Sidak's *post hoc* test	1	15%	17%
h	4	B	Frontal Cortex progranulin	Student's *t* test	1	15%	17%
i	4	C	Thalamus progranulin	Student's *t* test	1	8%	9%
j	4	D	Hippocampus progranulin	Student's *t* test	1	14%	16%
k	5	B	Frontal Cortex progranulin	Two-way ANOVA, Sidak's *post hoc* test	0.35	47%	54%
l	5	C	Hippocampus progranulin	Two-way ANOVA, Sidak's *post hoc* test	0.52	37%	42%
**Exercise-related positive controls**							
m	1	B	Hippocampus BDNF	Student's *t* test	0.57	35%	39%
n	1	B	Hippocampus α-tubulin	Student's *t* test	1	9%	10%
o	2	A	Running distance	Two-way Repeated Measures ANOVA	0.50	2092 m (38% change)	2360 m (43% change)
p	2	B	Body weight	Two-way ANOVA, Sidak's *post hoc* test	0.97	6.5 g (22% change)	7.3 g (25% change)
q	3	E	Hippocampus BDNF	Two-way ANOVA, Sidak's *post hoc* test	0.56	35%	40%
r	3	F	Hippocampus α-tubulin	Two-way ANOVA, Sidak's *post hoc* test	0.84	25%	29%
s	3	G	Dentate gyrus doublecortin-positive neurons	Two-way ANOVA, Sidak's *post hoc* test	0.38	8.3 cells (45% change)	9.4 cells (51% change)
t	4	E	Dentate gyrus doublecortin-positive neurons	Student's *t* test	0.10	33.9 cells (113% increase)	38.3 cells (127% increase)
u	4	D	Hippocampus BDNF	Two-way ANOVA, Sidak's post-hoc test	0.85	25%	28%
v	5	E	Hippocampus α-tubulin	Two-way ANOVA, Sidak's post-hoc test	1	7%	8%

## Results

### Effect of Exercise on Brain and Plasma Progranulin Levels in Young Mice

Prior work has shown that young mice (age 2–3 months), engage in more wheel running and experience greater neurotrophic and behavioral effects of exercise than older mice (age 15 months and older; Adlard et al., 2005a; van Praag et al., 2005). We therefore reasoned that exercise might be most likely to increase progranulin in young mice. We tested this hypothesis by housing 2-month-old wild-type mice with or without running wheels for 3–4 weeks. Mice with running wheels ran approximately 8 km/day (Fig. 1*A*), which was sufficient to produce a robust increase in hippocampal BDNF (Fig. 1*B*–*C* ^m,n^), confirming that the mice exercised enough to produce previously described neurotrophic effects. However, we did not observe an increase in frontal cortex (Fig. 1*D* ^a^) progranulin protein, and only a small increase in hippocampus (Fig. 1*E* ^b^). Plasma progranulin protein levels were also unchanged (Fig. 4*F* ^c^). A prior study (Asakura et al., 2011) had observed a larger (>100%) increase in hippocampal progranulin under similar conditions but used only *n* = 4, so our larger sample (*n* = 13–16) may provide a more reliable estimate. Power analysis using the observed variability indicated that we were powered to see an increase as small as 18% (see Table 1).

**Table 2:** Measures of Gliosis

Row	Figure	Panel	Description	Test	Power for 30% effect size	Power to normalize pathology	Delta at 70% power	Delta at 80% power
			Global CD68 analysis	Three-way ANOVA				
w	6	A	Frontal cortex CD68	Two-way ANOVA, Sidak's *post hoc* test	0.54	1	0.45% area(36% change)	0.51% area(41% change)
x	6	A	Hippocampus CD68	Two-way ANOVA, 's *post hoc* test	0.16	0.55	1.40% area (78% change)	1.58% area (88% change)
y	6	A	Thalamus CD68	Two-way ANOVA, Sidak's *post hoc* test	0.23	0.94	2.61% area (63% change)	2.95% area (71% change)

### Lack of Exercise Effect on Brain and Plasma Progranulin Levels in *Grn^+/−^* mice

**Figure 2:**
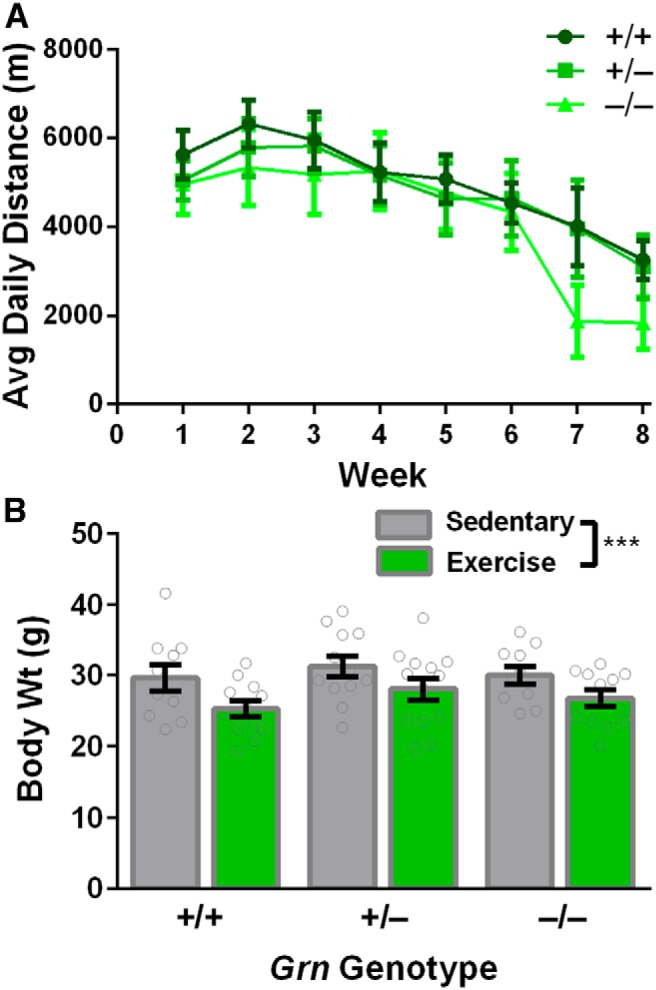
Progranulin deficiency does not affect wheel-running behavior. Mice aged 6 months were randomized to sedentary or exercise groups for 7.5 weeks. ***A***, No genotype differences were detected in wheel running distance between *Grn^+/+^*, *Grn^+/−^*, and *Grn^−/−^* mice. ***B***, Exercise reduced body weight similarly in all three genotypes (***ANOVA exercise effect, *p* = 0.003). *n* = 10–15 mice per group.

In FTD, any benefit of exercise on progranulin levels would need to occur in haploinsufficient individuals and at older ages, so we considered it important to examine exercise in *Grn^+/−^* mice at a symptomatic age. We used 6-month-old mice, around the earliest age at which we reliably detect behavioral effects of progranulin insufficiency (Filiano et al., 2013). To test the hypothesis that exercise could increase brain progranulin levels in *Grn^+/−^* mice, we solo-housed 6-month-old wild-type (*Grn^+/+^*), *Grn^+/−^*, and *Grn^−/−^* mice in standard mouse cages or cages with running wheels for 7.5 weeks. This running duration is in the range previously reported to exert neurotrophic effects in the hippocampus and to provide benefits in other mouse models of neurodegenerative disorders (van Praag et al., 1999a,b; Berchtold et al., 2005; Stranahan et al., 2012; Intlekofer and Cotman, 2013). The exercise group was given free access to running wheels over the testing period. No genotype difference in running distance was detected (Fig. 2*A* ^o^). Exercise significantly reduced body weight across genotypes (Fig. 2*B* ^p^), and there were no genotype differences in baseline body weight or in the effect of exercise on body weight.

**Figure 3:**
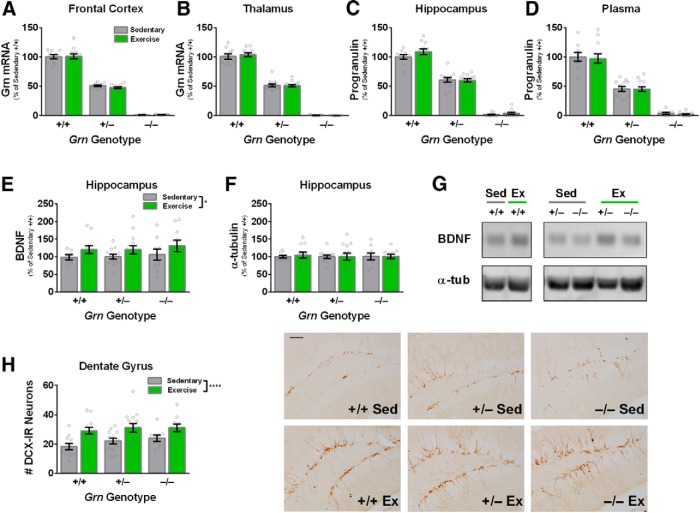
Exercise (7.5 weeks) does not increase progranulin in wild-type or *Grn^+/−^* mice. Exercise for 7.5 weeks did not increase progranulin mRNA in frontal cortex (***A***) or thalamus (***B***), or progranulin protein levels in the hippocampus (***C***) or plasma (***D***). ***E–G***, In contrast, exercise increased hippocampal BDNF levels across all three genotypes (***E***, ANOVA effect of exercise, *p* = 0.035), with no change in α-tubulin as a loading control (***F***). ***G***, Representative BDNF and α-tubulin blots. ***H***, Exercise also increased the number of doublecortin (DCX)-positive neurons in the dentate gyrus (ANOVA effect of exercise *p* < 0.0001, Sidak’s *post hoc* test exercise > sedentary *Grn^+/+^*, *p* = 0.0089, *Grn^+/−^*, *p* = 0.0198). Representative images (20×) are shown of doublecortin immunostaining in the dentate gyrus. Scale bar, 25 µm. Mice were 6-months-old on average when beginning the study, and 8-months-old on average when samples were collected. *n* = 10–15 mice per group. Values in ***A–F*** are expressed relative to the wild-type sedentary group.

Next, we tested the effects of exercise on progranulin levels. Wheel running for 7.5 weeks did not significantly increase any measure of progranulin in wild-type or *Grn^+/−^* mice. No effect was observed on progranulin mRNA levels in frontal cortex (Fig. 3*A* ^d^) or thalamus (Fig. 3*B* ^e^), or on progranulin protein levels in the hippocampus (Fig. 3*C* ^f^) or plasma (Fig.3*D* ^g^). Power analysis using the observed variability indicated that we were well powered to see increases in brain progranulin as small as 5–10% (see Table 1). Although it is uncertain how much brain progranulin would need to increase for a therapeutic effect, reaching the lower limit of normal, which is commonly defined as the fifth percentile of the control group (2 SDs below the control group mean), is a reasonable estimate. Because the SD of brain progranulin in wild-type mice was about 10% of the mean level, progranulin levels in *Grn^+/−^* mice would need to increase from 50% of normal to 80% of normal, i.e. by 30% of the normal value, to reach the lower limit of normal. Power analysis indicated that we had essentially 100% power to detect a change of this magnitude (see Table 1).

As positive controls, we measured hippocampal BDNF and neurogenesis. Hippocampal BDNF levels were increased to a similar extent in wild-type, *Grn^+/−^*, and *Grn^−/−^* mice (Fig. 3*E*–*G* ^q,r^). We measured neurogenesis by counting the number of doublecortin-positive neurons in the dentate gyrus. Exercise increased the number of doublecortin-positive neurons, indicating increased neurogenesis across all three genotypes (Fig.3*H* ^s^). There were no significant differences in the number of doublecortin-positive neurons between genotypes, nor was there a significant interaction of genotype and exercise.

**Figure 4:**
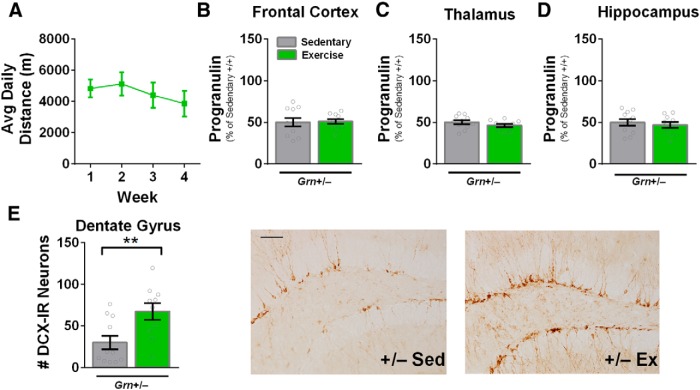
Exercise (4 weeks) does not increase progranulin protein levels in multiple brain regions of *Grn^+/−^* mice. Four- to 8-month-old *Grn^+/−^* mice were randomized to sedentary or exercise groups for 4 weeks (***A***). Exercise did not increase progranulin protein levels in frontal cortex (***B***), thalamus (***C***), or hippocampus (***D***), despite producing the expected increase in the number of doublecortin-positive neurons in the dentate gyrus (***E***, ** *p* < 0.01). Values in ***B***–***D*** are expressed relative to sedentary *Grn^+/+^* mice, with sedentary *Grn^+/−^* mice set at 0.5 to maintain a consistent scale with Figure 3. *n* = 11–12 per group.

After failing to observe an increase in hippocampal progranulin protein or cortical or thalamic progranulin mRNA in wild-type or *Grn^+/−^* mice with 7.5 weeks of exercise, we sought to confirm this negative result by testing a different duration of exercise and measuring progranulin protein in all three brain regions. The lack of change in progranulin mRNA in the cortex and thalamus indicates that exercise did not increase progranulin expression, but there could potentially be alterations in progranulin cleavage and degradation that could increase protein levels in these regions. We therefore tested 4- to 8-month-old *Grn^+/−^* mice with 4 weeks of wheel running (Fig. 4*A*), a time period that was previously reported to increase progranulin in wild-type mice (Asakura et al., 2011). Only *Grn^+/−^* mice were included in this group because the primary goal of this experiment was to determine if exercise were capable of increasing brain progranulin levels in *Grn^+/−^* mice. There was no significant increase in progranulin protein levels in the frontal cortex (Fig. 4*B* ^h^), thalamus (Fig. 4*C* ^i^), or hippocampus (Fig. 4*D* ^j^), despite observing the expected increase in doublecortin-positive neurons in the dentate gyrus (Fig. 4*E* ^t^), indicating increased neurogenesis as a positive control. Power analysis using the observed variability indicated that we were fully powered to detect changes in the range of 30%, and highly powered to detect changes in the 10–15% range (see Table 1).

**Figure 5:**
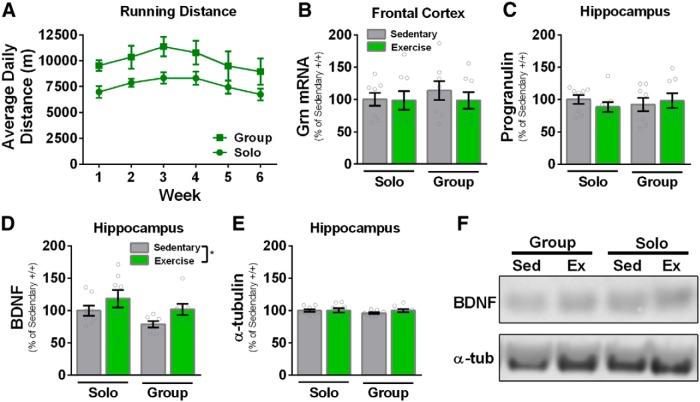
Group housing does not potentiate the effects of exercise on progranulin. Six weeks of wheel running (***A***) failed to increase frontal cortex progranulin mRNA (***B***) or hippocampal progranulin protein (***C***) in either solo- or group-housed wild-type mice aged 3–6 months. ***D-F***, Hippocampal BDNF was increased by exercise (***D***; ANOVA effect of exercise, *p* = 0.041). ***F***, Representative BDNF and α-tubulin blots for each group. *n* = 8–14 mice per group. Values in ***B***–***E*** are expressed relative to the solo-housed sedentary group.

We conducted an exploratory analysis to determine whether solo-housing could also have contributed to the lack of exercise effect on brain progranulin levels in 4- to 8-month-old mice. Solo-housing allows accurate measurement of wheel running by each mouse and ensures equal access to the running wheel for all mice. However, solo-housing can alter the neurotrophic and behavioral effects of exercise in rodents (Stranahan et al., 2006; Leasure and Decker, 2009; Hatchard et al., 2014). We therefore hypothesized that exercise in group-housed mice might increase brain progranulin. We tested this hypothesis with 3–6-month-old male and female wild-type mice. The mice were either solo- or group-housed (2–3 mice per cage) in standard or exercise cages for 6 weeks (Fig. 5*A*). There was no effect of solo- versus group-housing on progranulin mRNA in the cortex or progranulin protein in the hippocampus (Fig. 5*B*,C^k,l^). Whether solo- or group-housed, exercise had no effect on frontal cortical *Grn* mRNA levels (Fig. 5*B* ^k^), or hippocampal progranulin protein levels (Fig. 5*C* ^l^) despite producing significant increases in hippocampal BDNF (Fig. 5*D*–*F* ^u,v^). Although this exploratory analysis was less well powered than our primary analyses (Figs. 3, 4), the experiment was more likely than not to detect a 30% increase in hippocampal progranulin, and well powered to detect an increase of ∼40% in hippocampal progranulin (see Table 1). The experiment was less highly powered to detect a change in frontal cortex *Grn* mRNA with exercise in group-housing, but the trend was actually toward a small reduction in progranulin with exercise in this group (Fig. 4*B*). Overall, these results do not suggest that exercise is likely to have a greater effect on progranulin in group-housed mice.

Together, these experiments show that multiple exercise protocols sufficient to increase hippocampal BDNF and neurogenesis do not meaningfully increase progranulin in *Grn^+/−^*mice. These studies were sufficiently powered to detect even small changes in progranulin levels (see Table 1).

### Reduced Cortical Microgliosis in *Grn^−/−^* Mice

**Figure 6:**
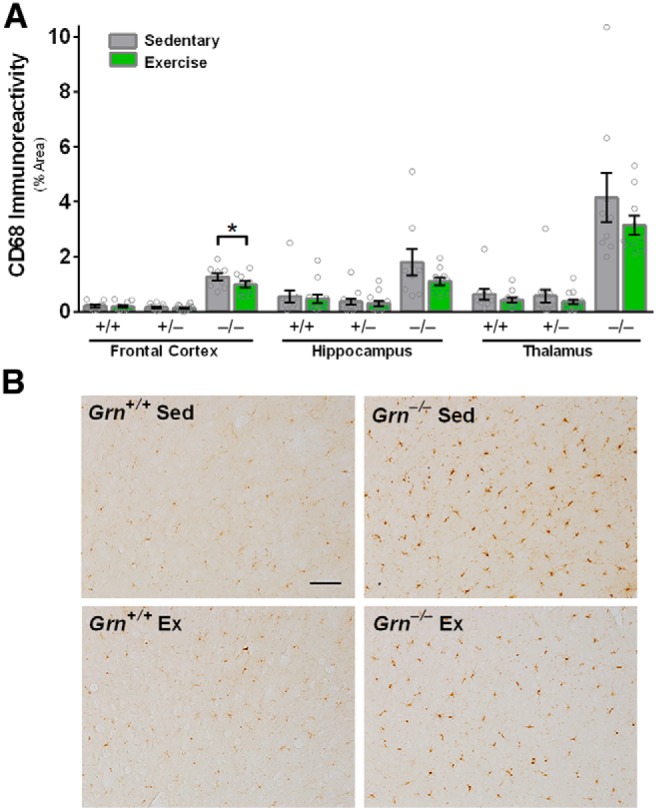
Exercise (7.5 weeks) reduces cortical microgliosis in *Grn^−/−^* mice. ***A****, Grn^−/−^* mice had elevated CD68 immunoreactivity in the frontal cortex, hippocampus, and thalamus (ANOVA genotype effect, *p* < 0.0001). Global analysis of CD68 immunostaining revealed a region x genotype interaction (*p* < 0.0001), so each region was analyzed with a separate ANOVA. Analysis of CD68 immunostaining in the frontal cortex revealed an effect of genotype (ANOVA, *p* < 0.0001) and a strong trend for an exercise effect (ANOVA exercise effect, *p* = 0.051). Subsequent *post hoc* analysis revealed significantly lower CD68 immunoreactivity in exercised relative to sedentary *Grn^−/−^* mice (*p < 0.05). ***B***, Representative images (20×) of CD68 immunostaining from the frontal cortex. Scale bar, 25 µm.

Although it was not the primary endpoint of our analyses, we took the opportunity to assess whether exercise, which is known to have anti-inflammatory effects, might reduce gliosis and inflammation in *Grn^−/−^* mice, which would be independent of an effect on progranulin levels. We assessed microgliosis by immunostaining for CD68 and found significantly elevated CD68 immunoreactivity in the frontal cortex, hippocampus, and thalamus of *Grn^−/−^* mice, as expected (Fig. 6*A* ^w-y^). There were trends toward a beneficial effect of exercise in all three regions, reaching significance on *post hoc* testing after correction for multiple comparisons only in frontal cortex. We also assessed inflammation in frontal cortex by measuring levels of the proinflammatory genes *Tnfa*, *Mcp1*, and *Il6*. We observed no significant effect of genotype for any of these genes (data not shown), indicating that 7- to 8-month-old *Grn^−/−^* mice have not yet developed the elevated levels of inflammatory cytokines found at older ages (Filiano et al., 2013). Similar results were obtained with GFAP staining for astrocytosis; there were significant increases in GFAP immunoreactivity in *Grn^−/−^* mice at this age, but they were small and variable enough that we were not well powered to detect reduction by exercise. *Grn^−/−^* mice also exhibited the expected accumulation of autofluorescent lipofuscin granules, which were also present at equivalent levels after exercise, but this phenotype was variable enough that we were not highly powered to detect exercise effects. Further study of older mice with greater pathology will be necessary to evaluate the effects of exercise on these neuropathological features in *Grn^−/−^*mice.

## Discussion

In this study, we investigated whether physical exercise could increase progranulin levels and thus ameliorate progranulin deficiency in *Grn^+/−^*mice, a model of FTD due to *GRN* mutations. We found that exercise did not produce sufficient increases in progranulin mRNA or protein levels in hippocampus, frontal cortex, or thalamus of 4- to 8-month-old *Grn^+/−^*mice under conditions that were sufficient to increase hippocampal BDNF and activate neurogenesis. Exercise did reduce cortical microgliosis in *Grn^−/−^* mice, showing a progranulin-independent anti-inflammatory effect of exercise.

This study does not support the idea of exercise as a strategy to raise brain progranulin levels in symptomatic patients with FTD due to *GRN* mutations, as we observed no effects on progranulin levels in *Grn^+/−^*mice at 4–8 months of age, the age range in which these mice begin to develop abnormal behavior and neuronal dysfunction (Filiano et al., 2013). These experiments were adequately powered to detect even minor increases in progranulin, and argue against the potential for progranulin-mediated benefits of exercise in *Grn^+/−^*mice at this age. Exercise did have modest effects on hippocampal (but not cortical) progranulin in very young wild-type mice (Fig. 1). The findings thus provide preliminary evidence for an interaction between age and exercise in modulating progranulin expression, with effects of exercise only in very young mice. The lack of effect of exercise on progranulin in older mice is similar to the pattern of exercise effects on hippocampal BDNF, which also decrease with age (Adlard et al., 2005a; van Praag et al., 2005). Greater running distance in the 2- to 3-month-old mice may partially explain these data, but the solo-housed wild-type mice used to test solo- versus group-housing (Fig. 5) ran similar distances as the young mice, yet did not show any increase in brain progranulin levels. Although we cannot rule out that exercise might also increase hippocampal progranulin in 2- to 3-month-old *Grn^+/−^* mice, there is no data that an increase of this magnitude (only 10–15%) restricted to hippocampus would be sufficient to produce functional improvement, and the fact that the effect is not sustained even until ∼4 months of age makes it highly unlikely to yield any sustained benefit after onset of functional changes between 6 and 12 months.

There could be, of course, other conditions under which exercise would increase progranulin expression. For example, longer duration of exercise or higher intensity exercise might produce an effect. In some studies of AD animal models, 5–6 months of exercise produced greater improvements in behavior or reductions in Aβ than 1 month of exercise (Adlard et al., 2005b; García-Mesa et al., 2011). However, in both of those studies, the 1 month exercise groups had either significant effects on behavior or clear, but not statistically significant, trends for reduction of Aβ. On the contrary, we did not observe promising trends for normalization of progranulin levels in 4- to 8-month-old *Grn^+/−^*mice that would prompt longer duration exercise studies.

We found a modest but significant reduction of cortical microgliosis in *Grn^−/−^* mice. There were similar trends for reduction of microgliosis in the hippocampus and thalamus that the study was likely underpowered to detect (Table 1, rows x, y). The reduction in CD68 immunoreactivity between sedentary and exercised *Grn^−/−^* mice is consistent with the general anti-inflammatory effects of exercise, including those found in other mouse models of neurodegenerative disease (Gleeson et al., 2011; Stranahan et al., 2012). We were unable to confirm the anti-inflammatory effects of exercise in the frontal cortex using inflammatory gene expression, as the *Grn^−/−^* mice used in this study (∼8 months when brains were collected) were not old enough to manifest these changes, which become prominent around 1 year of age (Ahmed et al., 2010; Wils et al., 2012).

In conclusion, we did not find evidence of exercise-stimulated increases in progranulin that would suggest a particular benefit of exercise for *GRN* mutation carriers. Although patients with *GRN* mutations should probably still be encouraged to exercise for its overall health benefits, including its anti-inflammatory effects, our data indicate that exercise is not a promising strategy for directly targeting progranulin deficiency.

## Synthesis

The decision was a result of the Reviewing Editor Jorge J. Palop and the peer reviewers coming together and discussing their recommendations until a consensus was reached. A fact-based synthesis statement explaining their decision and outlining what is needed to prepare a revision is listed below.

During the first round of revisions, Reviewer#1 accepted the manuscript and Reviewer#2 rejected and asked for a new cohort of GRN+/- mice, new measures of GRN levels, and methodological clarifications, which were implemented in the revised version. An additional Reviewer#3 reviewed the revised version and accepted it with some suggestions.

During the second round of revisions, Reviewer#2 and Reviewer #3 raised new concerns regarding effect sizes for power calculations. Instead of effect sizes of 100% for proganulin levels, we ask you to provide effect sizes of 20-30% change for power calculations in progranulin brain levels for Figure 3 to determine whether the power value is >0.70. We also think that it would be more informative to present what expected effect size the experimental design would be able to detect with a power of 0.7-0.8. After that, we suggest to remove the experiments of progranulin levels that are well underpowered (P 0.5), and discuss the experimental value of the progranulin experiments with power between 0.5 and 0.7 in terms of % effect sizes and therapeutic value. You might want to describe the underpowered experiments in the text, but the readers need to be alerted about this caveat. In general, we think it is important to define better the power calculation table, indicating whether there are post-hoc or not and the effect size for each power value. A paragraph discussing power calculations for negative results might be also appropriate for this manuscript. Please add new columns to the power table and explain the methodology in the method section. As it is currently described, it is difficult to fully understand the methods used. The above comments are critical since it is important for validating the experimental power of the negative results and, therefore, the main conclusion of the manuscript. Finally, we also feel that Fig. 1 has very little value since does not contain positive controls or GRN+/- mice or positive controls.

### Response to Reviewer Concerns

We appreciate the reviewers' constructive feedback. The majority of reviewer concerns concerned power analyses for the negative data on progranulin levels. As described below, we have adopted the reviewer suggestions regarding power analysis and have incorporated them into the revised manuscript.

1. "Instead of effect sizes of 100% for progranulin levels, we ask you to provide effect sizes of 20-30% change for power calculations in progranulin brain levels for Figure 3 to determine whether the power value is 0.70. We also think that it would be more informative to present what expected effect size the experimental design would be able to detect with a power of 0.7-0.8."

We have performed new power analyses for all data as requested. We have added columns to the power analysis table showing the power of the study to detect a 30% increase in progranulin or positive control measures, and the effect size detected by a power of 0.7 and 0.8. For the pathology measures we added these columns and an additional column showing the power for the change needed to completely normalize pathology in Grn-/- mice.

We should emphasize that we are conservative on the power estimates, as everything is based on two-tailed analyses. Since we are only looking for an increase in progranulin, a one-tailed analysis could have been considered appropriate, and would further increase power, but we opted for the more conservative approach, since we are essentially 100% powered for all the primary outcome measures (Figs. 3 and 4) even with the two-tailed analysis.

2. "After that, we suggest to remove the experiments of progranulin levels that are well underpowered (P 0.5), and discuss the experimental value of the progranulin experiments with power between 0.5 and 0.7 in terms of % effect sizes and therapeutic value."

We revised the manuscript as requested by removing values for which there was not at least 50% power to detect a 30% increase in progranulin. As is described in further detail in the revised manuscript, we estimate that a 30% increase in progranulin is the change required to bring an average Grn+/- mouse within 2 standard deviations of wild-type levels, making this a reasonable estimate for a biologically significant increase. As a result of this new criteria, we have excluded the acute exercise progranulin experiment (old Fig. 1, also requested by the reviewers due to lack of positive controls), as well as plasma progranulin in Fig. 5. This more rigorous and detailed analysis confirmed that we were adequately powered for all measures of brain progranulin levels except for the frontal cortex progranulin levels in figure 5B, which if anything are trending toward a decrease in progranulin rather than an increase. We added a statement in the text of the results section with this caveat for figure 5B.

The experiments with young adult (2-3-month-old) mice met the above criteria, but were underpowered to detect small (around 10%) changes in progranulin. In previous versions of this manuscript, there was a trend (p = 0.11) for an increase of around 9% in the hippocampus of young adult mice (figure 1). This lead to concern that exercise might have a small, but significant effect on progranulin levels in the hippocampus of young adult mice. We therefore added more animals to this study to increase the power to detect such changes (n = 5 per group) and found that exercise does produce a small increase in hippocampal progranulin in young adult mice. It should be noted that insufficient power does not explain the lack of exercise response in 4-8-month-old Grn+/- mice, as all of these studies have 70% power to detect changes of 5-15% and show no trends for increases in progranulin with exercise.

For pathology measures, we applied a less stringent power threshold, as these measures were exploratory secondary outcomes not critical to our primary hypothesis that exercise would increase progranulin levels in Grn+/- mice. For these measures, we excluded values for which there was not power of at least 0.5 to detect normalization of pathology in Grn-/- mouse. Measures of GFAP and lipofuscinosis had power around or less than 0.5 in several brain regions and so the data are not included in the revised figures.

3. "A paragraph discussing power calculations for negative results might be also appropriate for this manuscript."

We have incorporated discussion of power calculations for the negative progranulin data throughout the results section to assist readers in interpreting the data.

4. "Please add new columns to the power table and explain the methodology in the method section."

We have updated the power table as described in response #1. We have also added a description of power analysis to the methods section
